# Factors Associated With Genotypic Resistance and Outcome Among Solid Organ Transplant Recipients With Refractory Cytomegalovirus Infection

**DOI:** 10.3389/ti.2023.11295

**Published:** 2023-06-15

**Authors:** Yanis Tamzali, V. Pourcher, L. Azoyan, N. Ouali, B. Barrou, F. Conti, G. Coutance, F. Gay, J. Tourret, D. Boutolleau

**Affiliations:** ^1^ Sorbonne Université, Paris, France; ^2^ Department of Infectious and Tropical Diseases, Assistance Publique—Hôpitaux de Paris, Hôpital Pitié-Salpêtrière, Paris, France; ^3^ Assistance Publique—Hôpitaux de Paris, Hôpital Pitié-Salpêtrière, Medicosurgical Unit of Kidney Transplantation, Paris, France; ^4^ INSERM UMR 1146, Paris, France; ^5^ Institut Pierre Louis d’Epidémiologie et de Santé Publique, INSERM UMR, Paris, France; ^6^ Department of Nephrology Unité SINRA, Assistance Publique—Hôpitaux de Paris, Hôpital Tenon, Paris, France; ^7^ INSERM UMR 1038, Paris, France; ^8^ Department of Hepatogastroenterlogy, Assistance Publique—Hôpitaux de Paris, Hôpital Pitié-Salpêtrière, Liver Transplantation Unit, Paris, France; ^9^ Department of Cardiosurgery, Assistance Publique—Hôpitaux de Paris, Hôpital Pitié-Salpêtrière, Fédération de Cardiologie, Paris, France; ^10^ Department of Parasitology and Mycology, Assistance Publique—Hôpitaux de Paris, Hôpital Pitié-Salpêtrière, Paris, France; ^11^ Department of Virology, Assistance Publique—Hôpitaux de Paris, Hôpital Pitié-Salpêtrière, Centre National de Référence Herpèsvirus (Laboratoire Associé), Paris, France

**Keywords:** risk factors, cytomegalovirus, solid organ transplantation, opportunistic infections, antiviral resistance

## Abstract

Genotypically resistant cytomegalovirus (CMV) infection is associated with increased morbi-mortality. We herein aimed at understanding the factors that predict CMV genotypic resistance in refractory infections and disease in the SOTR (Solid Organ Transplant Recipients) population, and the factors associated with outcomes. We included all SOTRs who were tested for CMV genotypic resistance for CMV refractory infection/disease over ten years in two centers. Eighty-one refractory patients were included, 26 with genotypically resistant infections (32%). Twenty-four of these genotypic profiles conferred resistance to ganciclovir (GCV) and 2 to GCV and cidofovir. Twenty-three patients presented a high level of GCV resistance. We found no resistance mutation to letermovir. Age (OR = 0.94 per year, IC95 [0.089–0.99]), a history of valganciclovir (VGCV) underdosing or of low plasma concentration (OR= 5.6, IC95 [1.69–20.7]), being on VGCV at infection onset (OR = 3.11, IC95 [1.18–5.32]) and the recipients’ CMV negative serostatus (OR = 3.40, IC95 [0.97–12.8]) were independently associated with CMV genotypic resistance. One year mortality was higher in the resistant CMV group (19.2 % *versu*s 3.6 %, *p* = 0.02). Antiviral drugs severe adverse effects were also independently associated with CMV genotypic resistance. CMV genotypic resistance to antivirals was independently associated with a younger age, exposure to low levels of GCV, the recipients’ negative serostatus, and presenting the infection on VGCV prophylaxis. This data is of importance, given that we also found a poorer outcome in the patients of the resistant group.

## Graphical Abstract

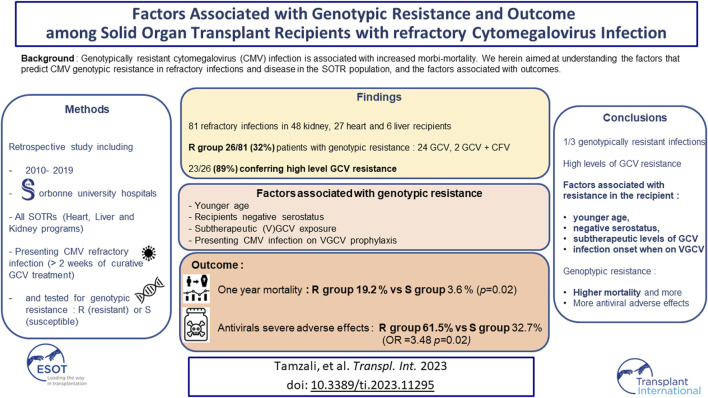

## Introduction

Cytomegalovirus (CMV) infection is the most common opportunistic infection in SOTRs (Solid Organ Transplant Recipients), occurring in 10%–40% of the patients, depending on the anti-CMV prophylaxis strategy [1–4]. The morbidity and mortality of CMV infection is now well-established, and its mechanisms more and more understood [1, 5–7].

The topic of ganciclovir (GCV)-resistant CMV infection has emerged in the last decades in the SOTR population [7], with a growing number of cases and an even increased morbi-mortality [5, 7–9]. Among some patients treated with GCV harboring a high viral load, CMV UL97 phosphotransferase mutated strains may emerge, conferring low to high level of resistance to the antiviral. When a low dose of GCV is persistently prescribed because of a low-level resistance, additional mutations in the UL54 DNA polymerase can be selected which confers high-level resistance to GCV and sometimes a cross-resistance to cidofovir (CDV) and foscarnet (FOS) [10]. High dose of GCV or second line antivirals (FOS, CDV) frequently lead to adverse effects, including cytopenia and kidney failure, both potentially affecting treatment success (due to early discontinuation or doses reduction) and impairing transplant and patients’ outcomes [5]. Nowadays, CMV resistance to antivirals is generally assessed with genotypic assays (UL97 and UL54 genes sequencing). This implies important technical work, can necessitate up to 2 weeks, sometimes resulting in an empiric dose increase or in a second line antiviral therapy switch. Therefore, understanding the determinants of CMV resistance and its consequences on outcome is critical, especially in SOTRs who already present comorbidities and possibly multiple medications.

We aimed at assessing risk factors for genotypically resistant CMV in the population of clinically refractory SOTRs and the factors associated with outcome (i.e., 1-year mortality and treatments adverse effects).

## Materials and Methods

### Study Design, Setting, and Participants

We performed a retrospective cohort study, by including all SOTRs (kidney, heart and liver transplant programs only in this University Hospital) with a CMV genotypic resistance testing for CMV refractory infection or disease (inclusion only if refractory, see further) between 1st January 2010 and 31st December 2019 in two centers (Pitié-Salpêtrière University Hospital and Tenon University Hospital, Assistance Publique-Hôpitaux de Paris, France).

The patients were divided in two groups according to the results of the resistance testing: susceptible (S) in case of the absence of resistance mutations and resistant (R) in case of the presence of at least one resistance mutation towards GCV, FOS or CDV.

Clinical biological, therapeutic data and outcome were collected from medical charts and the department of virology database. Data about follow-up was collected until 1-year after CMV infection onset.

### Clinical Data and CMV Definitions

CMV infection, disease and refractory infection were defined according to the current international recommendations on CMV management and definitions for use in clinical trials [11, 12].

Because of working in the context of refractoriness, CMV infection was defined as the positivity of one quantitative Polymerase Chain Reaction (PCR) on whole blood with a CMV load ≥3.0 log(IU/mL) using the artus^®^ CMV RGQ MDX kit (Qiagen) without any symptoms of the disease.

CMV disease was defined as CMV infection with any involvement of the following organs: eye, liver, digestive tract, bone marrow, lung, kidney, or central nervous system (CNS). Patients presenting with anomalies compatible with CMV syndrome or tissue invasive CMV disease were classified as CMV disease [13].

Refractory CMV infection was qualified in case of meeting the definition of a probable or certain CMV refractory infection as defined by Chemaly et al. [12] i.e., a persistent (<1.0 log decrease, above 1,000 IU/mL) or increased viral load after 14 days of appropriate antiviral treatment. Refractory CMV disease was qualified in case of meeting the definition of Chemaly et al [12]. of a probable or certain CMV disease, i.e., of a lack of improvement of the symptoms after 14 days of appropriate antiviral treatment.

Immunosuppression alleviation was defined as the discontinuation or reduction of 50% or more of the dose of one the following drugs: calcineurin inhibitors, antimetabolites, mammalian Target Of Rapamycin inhibitors and belatacept.

Valganciclovir (VGCV) underdosing was defined either as a daily dose ≤50% of the appropriate dose for more than a week, taking into account the estimated Glomerular Filtration Rate according to the GPR tool [14], or as a documented low GCV plasma concentration defined as a trough level <1 µg/mL when investigating refractoriness.

Serious adverse events were defined as per the food and drugs administration definition (www.fda.org). The cases were adjudicated by two independent clinicians based on chart review.

### CMV Prophylaxis, Infection Monitoring and Genotypic Antiviral Resistance Testing

All CMV seronegative transplant recipients receiving a graft from a CMV seropositive donor received oral VGCV prophylaxis for 6 months regardless the type of organ transplanted (no systematic screening until VGCV discontinuation). All CMV seropositive liver transplant recipients received systematic prophylaxis for 3 months (no systematic screening until VGCV discontinuation). CMV seropositive kidney transplant recipients could received a 3-month prophylaxis (no systematic screening until VGCV discontinuation) or be screened weekly for CMV replication for 3 months, then monthly until 1 year and every 2 months until 2 years after transplantation (according to the center). CMV seropositive heart transplant recipients were screened weekly for CMV replication for 3 months, then monthly until 1 year and every 2 months until 2 years after transplantation. All seronegative CMV recipients receiving grafts from a seronegative donor were routinely screened weekly for CMV replication for 3 months, then monthly until 1 year and every 2 months until 2 years after transplantation (no prophylaxis regardless the type of organ transplanted).

After transplantation, CMV replication was monitored at every visit with systematic CMV PCRs in whole blood for all SOTRs without ongoing VGCV prophylaxis for at least 1 year. In case of symptoms or biological anomalies suggesting potential CMV disease, patients were also tested for CMV replication in blood samples and possibly organ biopsies.

CMV DNA was quantified in clinical samples using the artus^®^ CMV RGQ MDX kit (Qiagen).

In case of refractory infection, GCV plasma trough concentration measuring and CMV genotypic resistance testing towards GCV, FOS, and CDV were performed by UL97 and UL54 gene sequencing, as previously described [6, 15].

We also performed retrospectively UL56 and UL89 gene sequencing for the screening of CMV resistance to letermovir (LMV), as previously described [16].

### Statistical Analysis

Quantitative variables are presented as mean ± standard deviation or median [IQR] according to their normal or skewed distribution. Comparisons were made using the Student’s t-test or the Mann Whitney Wilcoxon test according to their normal or skewed distribution. Qualitative variables are presented as numbers (percentages). The data were compared using the Fisher or Chi^2^ test.

We studied the association between genotypic resistance, 12-month mortality, antiviral toxicity and other collected variables. Risk factors for CMV resistance were searched using univariate logistic regression, including all characteristics differently distributed between the cases (CMV resistance to antivirals) and controls (CMV susceptibility to antivirals) (respectively the R and the S groups) with a *p*-value <0.1. A two-sided *p*-value <0.05 was considered statistically significant in the univariate analysis. The choice of the adjustment variables for multivariate analysis was made according to the existing literature, the results of the univariate analysis and the models with the lowest Akaike Information Criterion. The factors associated with 1-year mortality and treatment adverse effects were searched using the same method.

Survival analyses were performed using the Kaplan-Meier method. A Log-rank test was performed for the comparison between the two groups, with a *p*-value threshold at <0.05.

The statistical analysis was made using GraphPad PRISM^®^ (GraphPad Software, San Diego, CA, United States) and RStudio^®^ (R Software Boston, MA 02210).

### Ethics

All the patients undergoing healthcare at Assistance Publique-Hôpitaux de Paris agree to the retrospective use of their data by Assistance Publique-Hôpitaux de Paris healthcare providers for research purposes except if mentioning otherwise (http://eds.aphp.fr).

## Results

### Patients Population

Between 1st January 2010 and 31st December 2019, 3,711 patients received a solid organ transplant in Sorbonne University Hospitals, resulting in a cohort of 32,394 patients-years (patients with a functioning graft). CMV genotypic resistance testing was performed for 81 different SOTRs for refractory CMV infection or CMV disease in the two centers, including 48 kidney transplant recipients (59%), 27 heart transplant recipients (34%) and 6 liver transplant recipients (7%) (Table 1).

**TABLE 1:** Comparison of patient characteristics, CMV infection and outcome in the R (Resistant) and S (Suceptible) CMV groups.

	S group *N* = 55	R group *N* = 26	*p*
Clinical characteristics			
Age (years, mean ± SD)	54.8 ± 10.0	48.7 ± 12.2	0.02
Sex (male, *n*, %)	42 (76.4)	15 (57.7)	0.1
Transplanted organ (*n*, %)			0.9
Kidney	32 (58.2)	16 (61.5)	
Heart	19 (34.5)	8 (30.8)	
Liver	4 (7.3)	2 (7.7)	
Rank of transplantation (*n*, %)			
1	51 (92.7)	24 (92.3)	0.9
≥2	4 (7.3)	2 (7.7)	
CKD stage ≥ IV^a^ (*n*, %)	15 (27.3)	7 (26.9)	1
Induction with antithymocyte therapy (*n*, %)	47 (85.5)	23 (88.5)	1
Immunosuppressive regimen (*n*, %)			
Calcineurin Inhibitors	55 (100.0)	26 (100.0)	1
MMF	55 (100.0)	26 (100.0)	1
Corticosteroids	51 (92.7)	24 (92.3)	1
CMV serostatus and prevention			
CMV serostatus			
Donor negative, recipient negative (D−/R−), *n* (%)	2 (3.6)	1 (3.8)	
Donor positive, recipient negative (D+/R−), *n* (%)	9 (16.4)	13 (50.0)	0.002
Donor positive, recipient positive (D+/R+), *n* (%)	44 (80)	12 (46.2)	
Post-transplant CMV prevention strategy (*n*, %)			
Preemptive treatment	33 (60.0)	8 (30.8)	0.03
Prophylaxis	22 (40.0)	18 (69.2)	
VGCV prophylaxis underdosing or low plasma concentration^b^ (*n*, %)	13 (23.6)	16 (61.5)	0.002
CMV infection characteristics			
Post-transplant delay before CMV infection onset (months, median [IQR])	1 [0–5]	3 [1–5]	0.2
Patient on VGCV prophylaxis at CMV infection onset (*n*, %)	7 (12.7)	9 (34)	0.03
Peak viral load (UI/mL, median [IQR])	13,186 [3,132 32,147]	25,088 [9,422–153922]	0.02
CMV infection (*n*, %)	17 (31.0)	6 (23.0)	0.8
CMV disease (*n*, %)	38 (69.0)	20 (77.0)	
Organs involved in CMV disease (*n*, %)			
Bone marrow	25/38 (65.8)	20/20 (100.0)	0.002
Digestive tract (elevated LFTs, colitis)	22/38 (57.9)	15/20 (75.0)	0.25
Lungs	0/38 (0)	3/20 (15.0)	0.036
CNS	0/38 (0)	1/20 (5.0)	0.3
Other	0/38 (0)	2/20 (1kidney, 1 eye) (10.0)	0.1
Outcome			
Time for viral clearance (days, median [IQR])	50.0 [30.0, 97.5]	105.00 [67.0, 240.0]	<0.001
Antivirals serious adverse events^c^, (*n*, %)	18 (32.7)	16 (61.5)	0.03
One year mortality (*n*, %)	2 (3.6)	5 (19.2)	0.02^d^

CKD, chronic kidney disease; CNS, central nervous system; eGFR, estimated Glomerular Filtration Rate; LFTs, liver function tests; MMF, mycophenolate mofetil; VGCV, valganciclovir.

^a^ Defined by an estimated CKD EPI eGFR <30 mL/min/1.73 m^2^.

^b^ Underdosing was defined as a daily dose <50% of the recommended dose for eGFR according to http://sitegpr.com and low plasma concentration was defined as a Valganciclovir trough level < 1 µg/mL

^c^ Defined as the presence of at least one of the following events attributed to CMV, treatment among: cytopenia, acute kidney injury, elevated liver enzymes, neuropathy, mental status alterations, seizures.

^d^ Result by the Kaplan-Meier method/log rank test.

CMV resistance to at least one antiviral (GCV, FOS, CDV) was detected in about one-third of transplant recipients (26/81, 32%), Tables 1, 2.

**TABLE 2:** Mutations found in the genes of interest for CMV antiviral resistance.

UL97 phosphotransferase mutations (24)	UL54 DNA polymerase mutations (4)	UL56/UL89 terminase complex mutations (0)
L595S (10)		
A594V (4)		
C603W (3)		
L595F (2)	F412L (1)	
M460V (1)	K545S (1)	—
A594P (1)	K513N (1)	
Del 598-603 GKLTHC (1)	K545S (1)	
M460I and C603W (1)		
Del GKLTH 598-602 (1)		

The number of patients presenting the mutation is indicated within parentheses.

### Patients Characteristics and CMV Infection Presentation

The patients in the R group were younger (48.7 *versus* 58.8%, *p* = 0.022), more frequently CMV seronegative at the time of transplantation (53.8 *versus* 20.0%, *p* = 0.005), and presented more CMV primary infections than patients in the S group (53.8 *versus* 20.0%, *p* = 0.001). Three patients acquired primary infection when having CMV seronegative donors (one in the R and two in the S group). As expected, the peak viral load was significantly higher in the R group (25,088 [9,422–153,922] *versus* 13,186 [3,132–32,147] IU/mL, *p* = 0.02). Sixty-one percent of the patients in the R group presented a history of VGCV underdosing or a low plasma concentration *versus* 23.6% in the S group (*p* = 0.02) (Table 2)*.*

In the multivariate analysis, a younger age (OR = 0.94 per year, IC95 [0.089–0.99]), a history of VGCV underdosing or low plasma concentration (OR = 5.6, IC95 [1.69–20.7]), being on VGCV at infection onset (OR = 3.11, IC95 [1.18–5.32]) and the recipients’ CMV negative serostatus (OR = 3.40, IC95 [0.97–12.80]) were identified as risk factors for CMV genotypic resistance (Table 3).

**TABLE 3:** Factors associated with CMV genotypic resistance in multivariate analysis.

Variable	OR	IC 95%	*p*
Age	0.94	0.089–0.99	0.02
Systematic VGCV prophylaxis	1.35	0.33–5.36	0.67
On VGCV prophylaxis at infection onset	3.11	1.18–5.32	0.03
Recipient CMV negative serostatus	3.40	0.97–12.8	0.06
History of VGCV underdosing or low plasma concentration	5.61	1.69–20.7	0.006

CMV, cytomegalovirus; eGFR, estimated Glomerular Filtration Rate; VGCV, valganciclovir.

### CMV Resistance to Antivirals

Resistance mutations in CMV UL97 phosphotransferase and UL54 DNA polymerase found in the 26 patients of the R group are summarized in Table 2. Thirteen mutations were found in 26 patients. Two patients presented two mutations (A594V in UL97 and K545S in UL54 for one and M460I in UL97 and F412L in UL54 for the other). Twenty-four of these genotypic profiles conferred resistance to GCV and two conferred resistance to GCV and CDV. All patients but three presented a high level of GCV resistance, one presented a low level of GCV resistance (A594P in UL97) [10, 17, 18] and two presented mutations with undetermined level of resistance (Del GKLTH 598-602 and Del 598-603 GKLTHC in UL97) [11].

We also retrospectively sequenced the CMV UL56 and UL89 terminase complex genes and found no resistance mutation to LMV among the 45 patients with available samples. All patients were naive of LMV and maribavir (MBV).

### CMV Management

All patients with refractory non-resistant CMV infection (group S) were treated with oral VGCV or intravenous GCV except for two who were treated with FOS (given clinical refractoriness). The treatments in the R group were distributed as follows: fifteen patients received FOS only, five (V)GCV (three with low levels of GCV resistance and one with severe acute renal failure treated with VGCV and immunomodulation only with favorable outcome), two FOS + MBV, one MBV alone, one specific anti-CMV immunoglobulins, and one no antiviral treatment (immunosuppression [IS] reduction only).

In the S group, 38/53 (71.6%) patients with available information underwent IS alleviation *versus* 23/24 (95.8%) in the R group (*p* = 0.03). The detail about IS alleviation is displayed in Table 4.

**TABLE 4:** Immunosuppression alleviation to deal with CMV refractory infection.

Strategy	S group (*N* = 55)	R group (*N* = 26)	*p*
No change, (*n*, %)	17 (30.9)	2 (7.6)	0.01
MMF 50% dose reduction, (*n*, %)	26 (47.3)	8 (30.8)	
MMF discontinuation, (*n*, %)	10 (18.2)	14 (53.8)	
Switch MMF to mTORi, (*n*, %)	1 (1.8)	2 (7.6)	
Switch Tacrolimus to CsA and MMF 50% dose reduction, (*n*, %)	1 (1.8)	2 (7.6)	

CsA, ciclosporin A; MMF, mycophenolate mofetil; mTORi, mammalian target of rapamycin inhibitors.

### Outcome

Time to viral clearance was longer in the R group: 105 [67.00, 240.00] *versus* 50 [30.00, 97.50] days, (*p* < 0.001). Three patients in the R group (12%) presented persisting CMV DNAemia by the end of the 1-year follow-up *versus* none in the S group (*p* = 0.03).

Thirty-four patients developed serious anti-CMV treatment toxicity, 16 (61.5%) in the R group and 18 (32.7%) in the S group (*p* = 0.03). Toxicities were mainly represented by FOS-related acute kidney injury in the R group (11/16, 69%) and GCV-related cytopenia in the S group (16/18, 89%). CMV genotypic resistance was independently associated with antiviral drug toxicity (OR 3.48 IC95 [1.21–10.07], Table 5).

**TABLE 5:** Factors associated with antiviral treatment toxicity in multivariate analysis.

Variable	OR	IC 95%	*p*
Age	0.99	0.095–1.03	0.6
Systematic (V)GCV prophylaxis	0.74	0.26–1.97	0.6
eGFR<30 mL/min	1.70	0.60–4.89	0.3
CMV genotypic resistance	3.48	1.21–10.07	0.02

eGFR, estimated glomerular filtration rate; (V)GCV, valganciclovir or ganciclovir.

The overall 1-year mortality was 8.6% (7/81 patients). The mortality rate was significantly higher in the R group (19.2% *versus* 3.6%, *p* = 0.015). The Hazard Ratio for mortality in the R group was HR = 7.4, IC95 [1.5–37.5]).

## Discussion

This study focuses on clinically refractory CMV infection/disease in SOTRs, the factors associated with CMV genotypic resistance, and the outcomes, including antiviral drug toxicity and mortality. This cohort included twenty-five cases of genotypically proven resistant CMV infection in transplant recipients, and twice as many clinically refractory infections without resistance. This is one of the largest descriptive cohorts of this type [5–9, 19–21].

In this study, only one-third of the clinically refractory CMV infections were explained by genotypic resistance. All patients with resistant CMV but three presented high level of GCV resistance. Most of the patients presented UL97 isolated mutations, and some UL97 and UL54 mutations, in accordance with previous studies [5, 8]. In cases of critical CMV disease with suspicion of resistance, FOS treatment should be considered. MBV is an alternative recently approved by the Food and Drug Administration for refractory and resistant CMV infection [22] with a better tolerance profile [23].

All patients presented genotypically susceptible CMV to LMV. However, LMV treatment failures–explained by the emergence of LMV resistance in GCV-resistant CMV infections treated with LMV [24–26] - tend to suggest that this molecule should rather be used as a secondary prevention of GCV-resistant CMV infection in the SOTR population or in case of contraindications in certain patients, like severe neutropenia for instance.

Clinical refractoriness in the S group can be explained by i) inobservance of VGCV treatment, ii) subtherapeutic levels of (V)GCV due to malabsorption, under-prescription or renal function improvement or misevaluation iii) the inclusion criterion requiring 2 weeks of treatment which might be too short to define clinically refractory CMV infection. Subtherapeutic levels of (v)GCV can also lead to genotypic resistance development [11, 27, 28]. Some authors suggest that 3 weeks of treatment are necessary before CMV viremia shows significant decrease and recommend this delay before testing for CMV genotypic resistance [5, 19]. Refractoriness without genotypic mutation is partially understood. The inability of the immune system to clear viremia despite VGCV treatment is the main hypothetical mechanism [5, 7].

We found that both being on VGCV prophylaxis at infection onset and the exposure to low levels of VGCV were associated with CMV resistance: patients presenting refractory infection in this particular setting should be considered as more at risk of genotypically resistant infection. We also found significantly more patients with a history of VGCV prophylaxis in the R group: pre-emptive strategy could reduce resistance development through reduced GCV exposure [5, 6, 11], but CMV prophylaxis strategy did not result significative in multivariate analysis accordingly with the recent findings of Acquier et al. [29] in a cohort of kidney transplant recipients. It has been known for a few years that VGCV exposure *per se* is not a risk factor for infection but that a longer exposure is associated with resistance development [29, 30]. The threshold of exposure is usually recognized as 6 weeks [1] but a recent study found a threshold of 8 weeks of treatment with active replication as a risk factor [29] rather than prophylaxis itself or the cumulated time on prophylaxis and curative treatment Finally, the potential effect of VGCV exposure on resistance development should be balanced with the effects of more episodes of CMV replication in patients with pre-emptive strategies (immune exhaustion and opportunistic infections for instance).

Consistently with Fisher’s study [8] more than 50% of the patients with resistant CMV were seronegative before transplantation: this correlates both with longer VGCV exposure (a longer treatment is recommended [7, 11, 19]) and higher viral loads in the context of primary infection, two reported risk factors for resistance [5–7]. Resistance was also found associated with a younger age, consistently with previous data [31]: seronegative patients tend to be younger, as the risk of CMV seropositivity increases with age, and this association may result of a confusion bias. Younger or seronegative patients presenting refractory infection should be considered more at risk of genotypically resistant CMV infection.

CMV resistance was associated with a poorer outcome, including a fivefold higher 1-year mortality in this group, accordingly with previous studies [8]. CMV replication persisted three times as long in the R group than in the S group which may have resulted in either CMV-specific complications or in increased immune exhaustion [5, 32]. Both 3-month (early) and 1-year mortality were previously reported increased in resistant CMV infections, pleading for a direct attributable effect of the persistence of viral replication on mortality. Second-line antiviral treatment toxicities may also have played a role. Treatment serious adverse effects were found associated with resistance but are obviously not related to resistance itself but more probably to FOS toxicity mainly (65.6% vin the R group *versus* 4.6% in the S group): acute kidney injury being the main adverse effect in the resistant group and being a major factor of morbi-mortality in various infectious diseases, as it leads to therapeutic difficulties and specific complications [33, 34]. GCV higher dosing may also have played a role in these toxicities.

Another potential factor to explain poorer outcome in the R group may be immunosuppression alleviation, more frequent in this group, likely due to in the difficulty to achieve viral clearance in this group. It may have resulted in more organ rejection but we did not collect rejection events in our study.

This study shows some limitations: it is a small scaled retrospective study, comparing two very heterogenous groups of patients. We could not build solid multivariate models due to the small number of patients and events, which limits the applicability of these results. Also, because genotypic testing was only performed upon clinicians’ demand in case of the suspicion of a refractory infection rather than systematically, it is therefore plausible that resistance may have been underdiagnosed in this population. We believe it allows to show some factors associated with genotypic resistance to help the clinician identify patients at risk during the turnaround time of genotypic resistance testing. It also shows coherence with the previously published results on the topic.

In conclusion, this study mainly shows among SOTRs with CMV refractory infection an association of CMV genotypic resistance with the recipients negative serostatus, the exposure to low levels of VGCV and a younger age. On the other hand, GCV-resistant CMV infection is (in this cohort and in the literature) associated with increased (probably attributable) morbimortality. The recent evolutions in CMV antiviral strategies could make a difference in the prognosis of this infection.

## Data Availability

The raw data supporting the conclusion of this article will be made available by the authors, without undue reservation.
