# Supplementary material for: Factors Associated With Genotypic Resistance and Outcome Among Solid Organ Transplant Recipients With Refractory Cytomegalovirus Infection
Source: Transpl Int. 2023 Jun 15;36:11295. doi: 10.3389/ti.2023.11295 (PMC10307959; doi:10.3389/ti.2023.11295)
Supplement: Supplementary file 1 [file DataSheet1.docx]

|  | | | | |  |
| --- | --- | --- | --- | --- | --- |
| **Variable** | **N** | **OR^1^** | **95% CI^1^** | **p-value** | |
| Age | 81 | 0.95 | 0.91, 0.99 | 0.027 | |
| Sex | 81 |  |  |  | |
| Female |  | — | — |  | |
| Male |  | 0.42 | 0.15, 1.15 | 0.090 | |
| CKD ≥ IV | 81 |  |  |  | |
| No |  | — | — |  | |
| Yes |  | 0.98 | 0.33, 2.75 | 0.97 | |
| Transplanted organ | 81 |  |  |  | |
| Heart |  | — | — |  | |
| Kidney |  | 1.19 | 0.43, 3.41 | 0.74 | |
| Liver |  | 1.19 | 0.14, 7.48 | 0.86 | |
| Rank of transplantation ≥2 | 81 | 1.47 | 0.32, 6.18 | 0.58 | |
| Induction with antithymocyte therapy | 81 |  |  |  | |
| No |  | — | — |  | |
| Yes |  | 1.30 | 0.34, 6.38 | 0.71 | |
| Calcineurin inhibitors through levels ≥10 ng/mL | 81 |  |  |  | |
| No |  | — | — |  | |
| Yes |  | 0.59 | 0.15, 1.89 | 0.40 | |
| MMF dose ≥1500/day | 81 |  |  |  | |
| No |  | — | — |  | |
| Yes |  | 1.62 | 0.58, 5.06 | 0.38 | |
| Recipients CMV serostatus | 81 |  |  |  | |
| Negative |  | — | — |  | |
| Positive |  | 0.21 | 0.08, 0.58 | 0.003 | |
| Donor CMV serostatus | 81 |  |  |  | |
| Negative |  | — | — |  | |
| Positive |  | 0.94 | 0.09, 20.9 | 0.96 | |
| Primary infection | 81 |  |  |  | |
| No |  | — | — |  | |
| Yes |  | 5.96 | 2.13, 17.7 | <0.001 | |
| Prophylaxis_strategy | 81 |  |  |  | |
| Preemptive |  | — | — |  | |
| Systematic |  | 3.37 | 1.28, 9.51 | 0.016 | |
| VGCV underdosing | 81 |  |  |  | |
| No |  | — | — |  | |
| Yes |  | 5.17 | 1.93, 14.6 | 0.001 | |
| VGCV prophylaxis at infection onset  Viral load | 81  81 | 3.63  1.00 | 1.18, 11.07  1.00, 1.00 | 0.026  0.72 | |
| Treatment duration (days) | 72 | 1.03 | 0.99, 1.08 | 0.19 | |
| Time clearance (days) | 66 | 1.01 | 1.00, 1.02 | 0.009 | |

^OR: odds ratio; CI: confidence interval^

**Supplementary Table 1 – Univariate analysis of the factors associated with genotypic resistance**

| **Variable** | **N** | **OR^1^** | **95% CI^1^** | **p-value** |
| --- | --- | --- | --- | --- |
| Resistance | 81 | 6.31 | 1.25, 46.5 | 0.035 |

**Supplementary Table 2 – Univariate analysis of the factors associated with mortality**

^OR: odds ratio; CI: confidence interval^

| **Variable** | **N** | **OR^1^** | **95% CI^1^** | **p-value** |
| --- | --- | --- | --- | --- |
| Age | 81 | 0.98 | 0.94, 1.02 | 0.27 |
| Genotypic resistance | 81 | 3.29 | 1.27, 8.93 | 0.016 |
| CKD ≥ IV | 81 | 1.57 | 0.58, 4.24 | 0.37 |
| Prophylaxis strategy | 81 | 1.04 | 0.43, 2.53 | 0.92 |

**Supplementary Table 3 – Univariate analysis of the factors associated with treatment toxicity**

^OR: odds ratio; CI: confidence interval^
